# Cartilage tissue healing and regeneration based on biocompatible materials: a systematic review and bibliometric analysis from 1993 to 2022

**DOI:** 10.3389/fphar.2023.1276849

**Published:** 2024-01-04

**Authors:** Meng-Xuan Yao, Yi-Fan Zhang, Wei Liu, Hai-Cheng Wang, Chuan Ren, Yu-Qin Zhang, Tai-Long Shi, Wei Chen

**Affiliations:** ^1^ Department of Orthopaedic Surgery, The Third Hospital of Hebei Medical University, Shijiazhuang, Hebei, China; ^2^ Key Laboratory of Biomechanics of Hebei Province, Shijiazhuang, Hebei, China; ^3^ NHC Key Laboratory of Intelligent Orthopaedic Equipment, Shijiazhuang, Hebei, China; ^4^ Department of Pharmacy, Cangzhou People’s Hospital, Cangzhou, China

**Keywords:** bibliometric analysis, citespace, bibliometrix, cartilage, healing and regeneration, biocompatible materials

## Abstract

Cartilage, a type of connective tissue, plays a crucial role in supporting and cushioning the body, and damages or diseases affecting cartilage may result in pain and impaired joint function. In this regard, biocompatible materials are used in cartilage tissue healing and regeneration as scaffolds for new tissue growth, barriers to prevent infection and reduce inflammation, and deliver drugs or growth factors to the injury site. In this article, we perform a comprehensive bibliometric analysis of literature on cartilage tissue healing and regeneration based on biocompatible materials, including an overview of current research, identifying the most influential articles and authors, discussing prevailing topics and trends in this field, and summarizing future research directions.

## 1 Introduction

Cartilage is a fundamental type of connective tissue that furnishes vital support and cushioning for the body (Teixeira et al., 2020; Fu et al., 2021). It plays an indispensable role in various structures of the body, most notably in joints such as the knee, hip, elbow, shoulder, and ankle, as well as in the spine (Moutos et al., 2007; Teixeira et al., 2020). Cartilage tissue healing and regeneration is an area of research focused on restoring normal cartilage structure and function, with the main goal of reducing pain, restoring normal joint function and preventing further damage to joint tissues (Chen et al., 2022). Cartilage injuries, especially those involving articular cartilage, are prevalent among physically active individuals. Such injuries predominantly affect the knee but can manifest in other aforementioned joints (Matava et al., 2023). The underlying causes of cartilage damage are manifold, ranging from forceful impacts due to sports injuries or accidental falls to progressive wear and tear over extended periods of usage. Additionally, factors like repetitive minor impacts, inappropriate joint twisting under weight, and misalignment due to congenital anomalies or prior injuries can contribute significantly to such damage. When cartilage is injured or deteriorates, it leads to symptoms like joint pain, swelling, stiffness, and a sensation of grinding or clicking when the joint is in motion. For some, the ramifications can be severe, impeding daily activities such as walking, stair-climbing, bending, and kneeling. Moreover, the joint might feel unstable, may occasionally give way, or even lock during motion (Cronström et al., 2023; Persson et al., 2023). Notably, damaged or diseased cartilage culminates in pain, restricted mobility, and dysfunction (Lin et al., 2022; O'Shea et al., 2022). Articular cartilage defects are particularly concerning as they don't heal autonomously and are susceptible to progressing to osteoarthritis. This progression can, in turn, degrade joint functionality, leading to disability and diminished life quality (Khalili et al., 2022).

One of the promising solutions lies in the realm of biocompatible materials. Specifically engineered to safely and beneficially interact with the human anatomy, these materials find extensive application in medical treatments. Their spectrum ranges from synthetic to natural and from organic to inorganic categories (Liu et al., 2022; Khalili et al., 2022). Examples of biocompatible materials include polymers, ceramics, metals, composites, and biomaterials (Moutos et al., 2007; Fu et al., 2021; Liu W. et al., 2022; Khalili et al., 2022).

Given their pivotal role in cartilage tissue healing and regeneration, biocompatible materials act as scaffolds for novel tissue proliferation and tissue restoration (Fu et al., 2021; Lin et al., 2022). Their multifaceted benefits span from infection prevention and inflammation reduction (Johnson and García, 2015; Mieszkowska et al., 2020; Fetz and Bowlin, 2022; Gvaramia et al., 2022; Tu et al., 2022) to drug or growth factor delivery to enhance healing (Mancipe et al., 2021; Tu et al., 2022).

Research into utilizing these biocompatible materials for cartilage tissue regeneration is burgeoning. To truly grasp the strides made in this domain, a thorough examination of the extant literature is pivotal. This paper embarks on a bibliometric analysis journey, delving deep into the contemporary research milieu, spotlighting influential works and contributors, and elucidating emergent themes and trends, thereby charting potential future research trajectories.

## 2 Methods

### 2.1 Source of bibliometric data and search strategy

To identify relevant publications on cartilage tissue healing and regeneration using biocompatible materials, we searched the Web of Science (WOS) core collection database (SCI-EXPANDED, SSCI, A&HCI, CPCI-S, CPCI-SSH, ESCI, CCR-EXPANDED, and IC) by Clarivate Analytics in Philadelphia, PA, United States, which includes journals, books, patents, conference proceedings, and web resources (including free and open resources). The WOS database was selected because it offers a more precise categorization of document types than Scopus, and it provides a vast collection of articles containing comprehensive information, such as titles, author names, cited references, and other relevant data (Yeung, 2019). The WOS database also provides information about citations of scientific publications dating back to 1900 and encompasses all highly influential scientific journals (Hu and Steinberg, 2009; Martín-Martín et al., 2021). In addition, the WOS database has two major strengths: reference tracing and citation reporting, which allow researchers to conduct searches within leading academic journals and citation networks, thereby facilitating the powerful tracing of references and citations, particularly valuable for exploring research outputs in a specific field or area of study (Huang et al., 2021).

The search formula used in this study was as follows: #1 = {[(TS =(Biocompatible Materials OR Biocompatible Material OR Material, Biocompatible OR Biomaterials OR Biomaterial OR Bioartificial Materials OR Bioartificial Material OR Material, Bioartificial OR Hemocompatible Materials OR Hemocompatible Material OR Material, Hemocompatible)) OR TI =(Biocompatible Materials OR Biocompatible Material OR Material, Biocompatible OR Biomaterials OR Biomaterial OR Bioartificial Materials OR Bioartificial Material OR Material, Bioartificial OR Hemocompatible Materials OR Hemocompatible Material OR Material, Hemocompatible)] OR AB=(Biocompatible Materials OR Biocompatible Material OR Material, Biocompatible OR Biomaterials OR Biomaterial OR Bioartificial Materials OR Bioartificial Material OR Material, Bioartificial OR Hemocompatible Materials OR Hemocompatible Material OR Material, Hemocompatible)}; #2 = {[(TS=(heal OR healing)) OR TI=(heal OR healing)] OR AB=(heal OR healing)}; #3 = {[(TS=(regenerate OR regeneration)) OR TI=(regenerate OR regeneration)] OR AB=(regenerate OR regeneration)}; #4 = {[(TS=(“Cartilage*”)) OR TI=(“Cartilage*”)] OR AB=(“Cartilage*”)}; and #5 = [#1 AND (#2 OR #3) AND #4].

**FIGURE 1:**
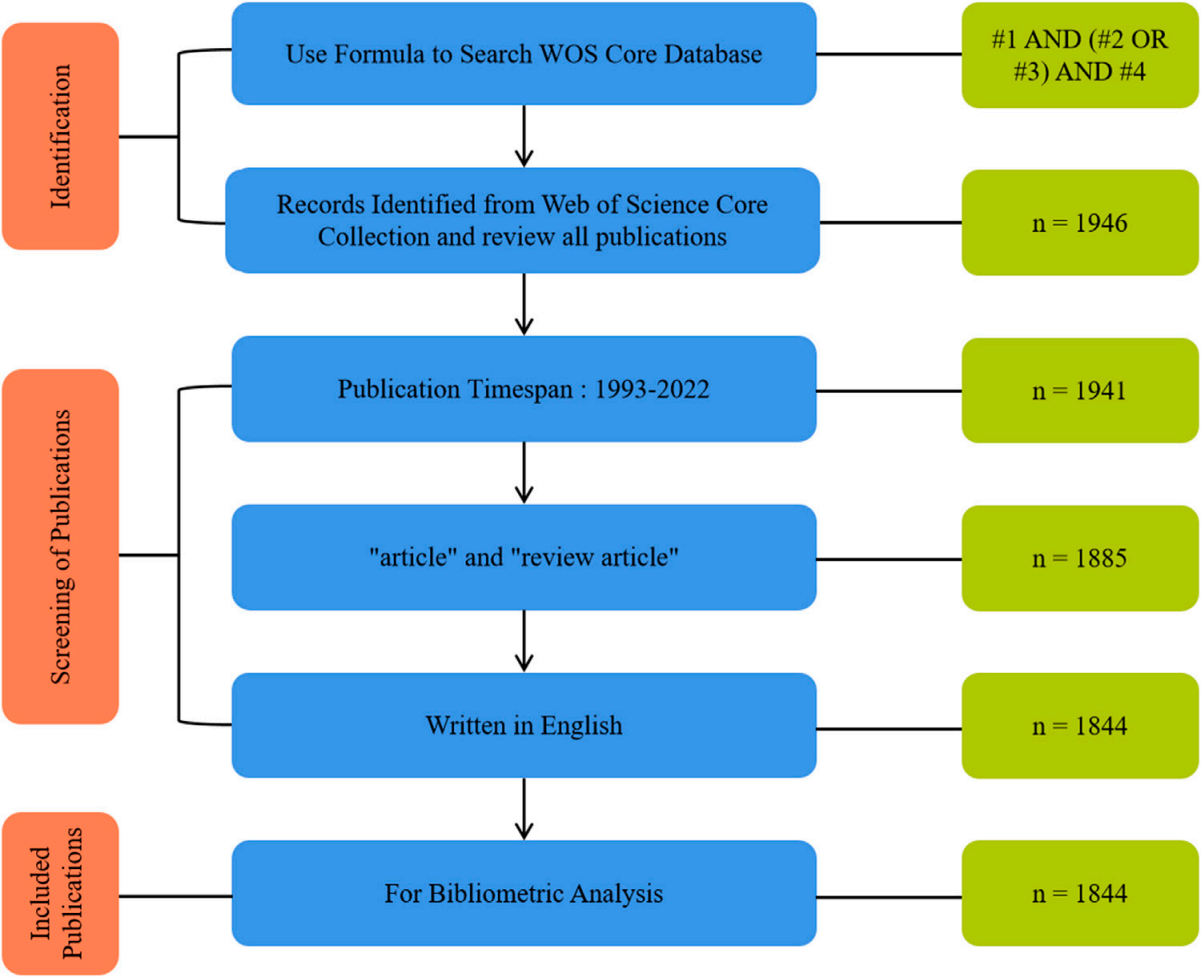
Flow chart of the search approach.

A cross-sectional search on December 7, 2022, using the search formula mentioned above, resulted in the retrieval of 1946 publications from the WOS database. All available published data were reviewed and assessed to identify publications specifically focusing on the healing and regeneration of cartilage tissue using biocompatible materials. Figure 1 outlines the search and inclusion/exclusion procedures for identifying relevant publications from the database. The literature search was restricted to English language publications published between 1993 and 2022. Only articles containing scientific information, such as research articles and reviews, were included (Van Leeuwen et al., 2003). After the screening process, the final results were exported to the dataset and included citation information such as author, document title, publication year, source title, volume, issue, page, citation count, source, and document type. Additionally, bibliographic information such as affiliation, edits, keywords and fund details were also included in the dataset. The full records and downloaded references of the retrieved articles were saved from the WOS database and stored in the.BibTeX format for further analysis.

Since the data used in this study were obtained from publicly available databases and did not involve any direct interaction with human or animal subjects, ethical approval was not necessary.

### 2.2 Analysis tools

To provide a comprehensive analysis of the literature on the healing and regeneration of cartilage tissue using biocompatible materials, all relevant data meeting the inclusion criteria were further analyzed using two software programs: Bibliometrix (RStudio, V1.4) (Aria and Cuccurullo, 2017) and CiteSpace V5.8 R3 (Drexel University, Philadelphia, PA, USA) (Mao et al., 2022).

### 2.3 Bibliometric analysis

The imported datasets were analyzed in the R package bibliometrix to generate line graphs representing publication trends by year. Furthermore, bibliometrix was utilized to analyze the annual publication trends, journals, countries and regions, cooperation relationships between countries/regions, and the number of citations. The top 100 high-frequency keywords were extracted using bibliometrix and visualized as word clouds and ThematicMaps. ThematicMap is a technique that involves creating a network of keywords and then generating typological themes for a domain in a two-dimensional map. The methodology is based on the proposal of Cobo et al. (Cobo et al., 2011) and allows easier interpretation of research topics formulated in the framework. The analysis is based on KeyWords Plus (KWP), a word or phrase frequently appearing in the title of the reference cited in the article but not in the title of the article itself. This feature is unique to the Clarivate Analytics database and helps to identify important keywords and topics related to a particular research area.

CiteSpace is a popular and widely recognized bibliometric visualization tool used to create collaborative maps between countries/regions, institutes, co-authors and reference co-authors and to calculate keyword outbreaks between 1993 and 2022. In this study, CiteSpace was used with the following format: time slicing from January 1993 to December 2022, with the option to choose the number of years per time slice for analyzing collaboration and keyword trends over time and identifying influential authors, institutions, and research groups in this field.

## 3 Results

### 3.1 General data information

Our search strategy resulted in a total of 1946 articles, of which 1844 met the inclusion criteria after further screening. Table 1 summarizes the general characteristics of all included articles. The total number of citations for all articles was 95,814, with an average of 51.96 citations per article. Of the included articles, 1,332 were research papers, accounting for 72.2% of all publications, and 512 were reviews, accounting for 27.8% of all publications. Overall, the field of healing and regeneration of cartilage tissue using biocompatible materials has attracted contributions from 67 countries/regions, 1992 institutions, 8,042 authors, and 503 journals.

**TABLE 1:** General data information.

Description	Results
Main information about the data	
Timespan	1993:2022
Sources (Journals)	503
Documents	1844
Annual Growth Rate %	19.63
Document Average Age	5.97
Average citations per doc	51.96
References	82,051
Document contents	
Keywords Plus (ID)	3,678
Author’s Keywords (DE)	3,084
Authors	
Authors	8,042
Authors of single-authored docs	40
Authors collaboration	
Single-authored docs	43
Co-Authors per Doc	6.29
International co-authorships %	26.25
Document types	
Article	1,332
Review	512

### 3.2 Publication trend

**FIGURE 2:**
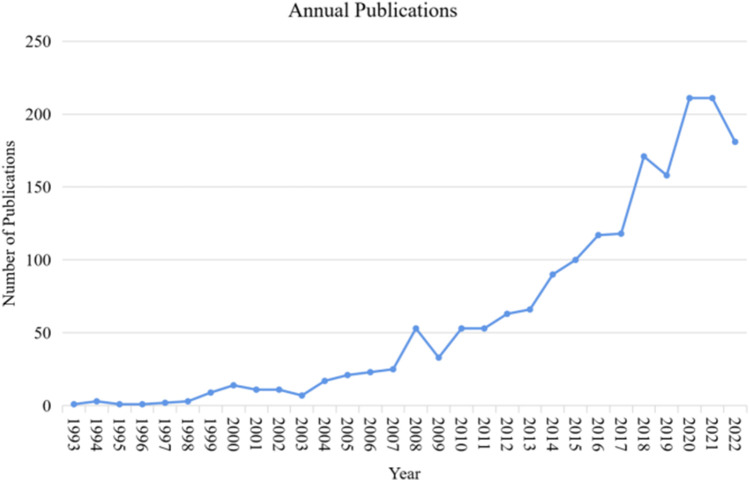
Publication trend from 1993 to 2022.

Figure 2 shows the number of all publications over time. We observed an overall upward trend, with an average annual growth rate of 19.63%. The trend can be divided into two stages: the first stage, from 1993 to 2007, during which the number of new publications did not exceed 50 per year, indicating a relatively stable. The second stage, from 2013 to 2022, showed the number of new publications exceeded 50 per year, except in 2009, and the overall trend was upward, except in 2019 and 2022 when the number of new publications was slightly lower than the previous year.

### 3.3 Countries/regions

**FIGURE 3:**
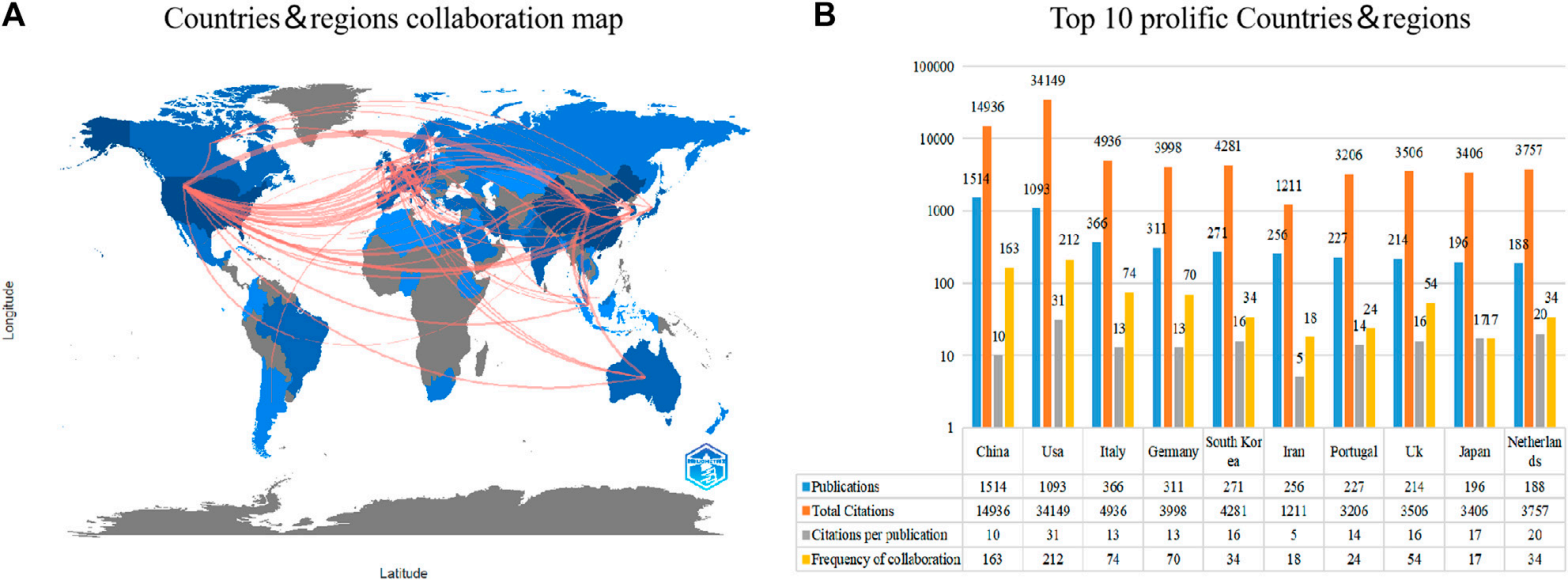
The top 10 most productive countries and international cooperative network. **(A)** Collaboration among prolific countries that published more than 10 papers. The depth of color represents the number of publications, with a darker shade indicating a higher number of publications. The width of the link is positively correlated with the strength of cooperation. **(B)** The number of publications, total citations (TC), citations per publication (CPP) and Frequency of collaboration (FOC) of the top 10 most productive countries/regions.

Figure 3A shows an international collaboration network consisting of countries/regions that have published at least 10 articles in the field of healing and regeneration of cartilage tissue using biocompatible materials. The countries with the highest number of published articles are China and the United States, which also actively collaborate with other countries/regions. Figure 3B lists the top 10 countries that have contributed the most to the field. China ranks first with 1,514 articles, followed by the United States with 1,093 articles and Italy with 366 articles. In terms of citations, the United States has the highest TCs (*n* = 34,149), CPPs (*n* = 31), and FOCs (*n* = 212). China ranks second in TCs (*n* = 14,936), second in FOCs (*n* = 163), and ninth in CPPs (*n* = 10). Italy ranks third in TCs (*n* = 4,936), third in FOCs (*n* = 74), and seventh in CPPs (*n* = 13).

### 3.4 Institutions

**FIGURE 4:**
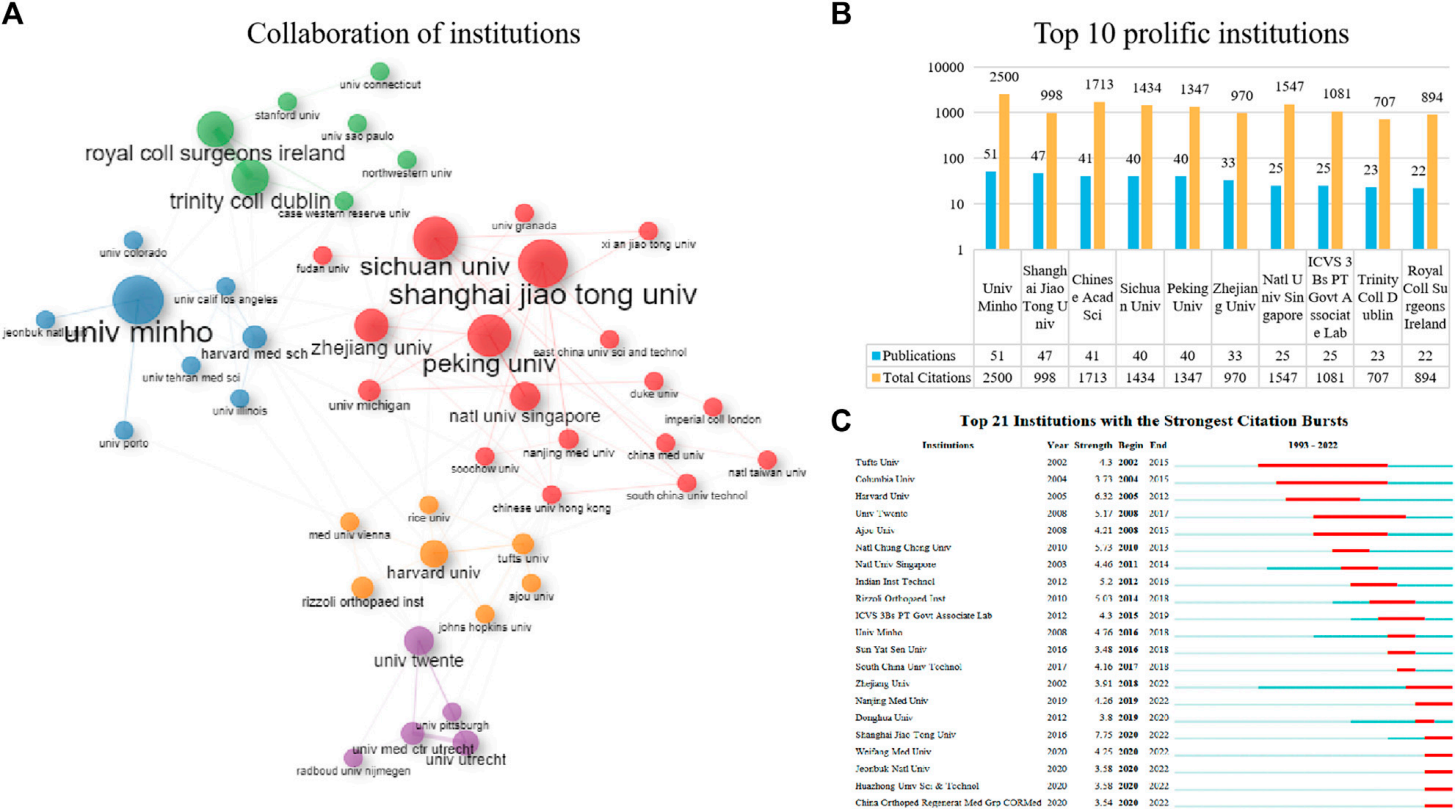
The top 10 most productive institutions and inter-institutions’ co-operative network. **(A)** Collaboration among these prolific institutions. **(B)** The number of publications, total citations (TC) in the top 10 prolific institutions. **(C)** Top 21 Institutions with the Strongest Citation Bursts.

Figure 4A presents the collaboration network among the top 50 institutions in terms of publication volume, with the two formed clusters indicating a close collaboration between them. The institutions belonging to the red cluster were mostly located in China, such as Shanghai Jiao Tong University, Sichuan University, and Peking University. Institutions belonging to the purple, green, blue, and orange clusters were mostly located in Europe and the United States, such as the University of Minho, Trinity College Dublin, Royal College of Surgeons in Ireland, and the University of Twente. Figure 4B lists the top ten contributing institutions in the field, with the University of Minho (51 publications) ranking first, followed by Shanghai Jiao Tong University (47 publications) and the Chinese Academy of Sciences (41 publications). In terms of citations, the University of Minho had the highest TC (*n* = 2,500), while the Chinese Academy of Sciences ranked second with a TC of 1713. Overall, among the top 10 highest productivity institutions, Chinese institutions had relatively more publications and citations compared to institutions from other countries. To visualize the timeline of activity of these institutions, we used the outbreak view in CiteSpace (Figure 4C). Tufts University, Columbia University, and Harvard University were active in the early period in this field, while the University of Minho, Sun Yat-Sen University, and the South China University of Technology became active after 2015. Since 2020, Chinese institutions such as Zhejiang University, Shanghai Jiao Tong University and Huazhong University of Science and Technology have been active in this field.

### 3.5 Journals

Table 2 presents the top 10 most productive journals and co-citation journals. The results showed that Acta Biomaterialia [IF (2021) = 10.633, Q1] was the most productive journal with 84 publications, followed by Biomaterials [IF (2021) = 15.304, Q1] with 74 publications, and the Journal of Biomedical Materials Research Part A [IF (2021) = 4.854, Q2] with 42 publications. In terms of citations and impact, Biomaterials ranked first (15,300 TC, 49 h index), followed by Acta Biomaterialia (5848 TC, 40 h index) and Journal of Biomedical Materials Research Part A (1637 TC, 21 h index). However, considering that the number of publications may not necessarily reflect their impact in a given field, CiteSpace was used to identify co-cited journals in this field, and the results indicated that the top three co-cited journals were Biomaterials, Acta Biomaterialia, and Journal of Biomedical Materials Research Part A. In terms of citations and impact, Biomaterials ranked first (15,300 TC, 49 h index), followed by Acta Biomaterialia (5848 TC, 40 h index) and Journal of Biomedical Materials Research Part A (1637 TC, 21 h index).

**TABLE 2:** Top 10 prolific journals and co-cited journals.

Journals	h_index	Publications	Total Citations	IF (2021)	JCR	Journal	Co-Cited	IF (2021)	JCR
Biomaterials	49	74	15300	15.304	Q1	Biomaterials	1629	15.304	Q1
Acta Biomaterialia	40	84	5846	10.633	Q1	Acta Biomater	1147	10.633	Q1
Journal Of Biomedical Materials Research Part A	21	42	1637	4.854	Q2	Journal Of Biomedical Materials Research Part A	1096	4.854	Q2
Biomacromolecules	20	22	1605	6.978	Q1	Tissue Engineering Part A	896	4.08	Q2
International Journal Of Biological Macromolecules	20	29	1101	8.025	Q1	Osteoarthritis and Cartilage	823	7.507	Q1
Journal Of Materials Chemistry B	19	31	854	7.571	Q1	Tissue Engineering Part A	799	4.08	Q2
Tissue Engineering Part A	19	36	1074	4.08	Q2	Biomacromolecules	727	6.978	Q1
Journal Of Tissue Engineering And Regenerative Medicine	16	28	1159	4.323	Q2	Science	710	63.714	Q1
ACS Applied Materials & Interfaces	14	22	1038	10.383	Q1	Proceedings Of the National Academy of Sciences of the United States of America	661	12.779	Q1
Advanced Functional Materials	14	17	827	19.924	Q1	Journal of Orthopaedic Research	649	3.102	Q2

### 3.7 Top cited articles

To identify the most influential research in this field, the Bibliometrix R Package was utilized to extract the top 10 locally most cited publications. Table 3 lists the top 10 most cited publications on cartilage tissue healing and regeneration, which comprised 2 original articles and 8 reviews. The locally most cited paper, with 84 citations, was “Tissue Engineering for Regeneration of Articular Cartilage,” published in 2000 in the journal Biomaterials by Temenoff J S et al. (Temenoff and Mikos, 2000), which introduced several commonly used tissue engineering techniques, including cell-based, matrix-based and biomaterial-based techniques. Cell-based techniques for cartilage regeneration involve the isolation and expansion of chondrocytes, which are the cells responsible for producing cartilage, from human or animal sources, while matrix-based techniques utilize biomaterials, such as collagen or hydroxyapatite, as scaffolds to support the growth of new cartilage tissues, and biomaterial-based techniques involve using materials that promote cell growth and differentiation to aid in the regeneration of cartilage tissues. The authors also discussed the challenges and limitations of tissue engineering techniques in regenerating cartilage, including the complexity of cell culture and matrix preparation, as well as the influence of *in vivo* biological and mechanical factors on cell growth and new tissue establishment. Moreover, the article also described potential future directions for tissue engineering techniques, including the use of biomaterials, gene therapy, and pluripotent stem cells to improve regeneration efficiency. The most cited paper worldwide (4,307 citations) is “Porous scaffold design for tissue engineering,” published in 2004 in the journal Biomaterials by Karageorgiou V et al. (Karageorgiou and Kaplan, 2005). This review article discussed the design and development of scaffolds for tissue engineering applications, focusing on aspects such as scaffold porosity, pore size, pore interconnectivity, and mechanical properties. The article also discussed the application of scaffolds in various tissue engineering contexts, including cartilage, bone, nerve and skin, the significance of scaffold design in tissue engineering, and the necessity for scaffolds mimicking the native extracellular matrix in terms of porosity, pore size, and mechanical properties.

**TABLE 3:** Top 10 cited publications.

Rank	Titles	Author	Document Type	DOI	Publication Year	Local Citations	Global Citations
1	Tissue engineering for regeneration of articular cartilage	Temenoff J S, Mikos A G	Review	10.1016/S0142-9612(99)00213-6	2000	84	831
2	Engineering cartilage tissue	Chung C, Burdick J A	Review	10.1016/j.addr. 2007.08.027	2008	76	521
3	Application of chitosan-based polysaccharide biomaterials in cartilage tissue engineering: a review	Suh JK, Matthew HW	Review	10.1016/s0142-9612(00)00126-5	2000	75	1579
4	Porosity of 3D biomaterial scaffolds and osteogenesis	Karageorgiou V, Kaplan D	Review	10.1016/j.biomaterials. 2005.02.002	2005	75	4307
5	Extracellular matrix scaffolds for cartilage and bone regeneration	Benders KE, van Weeren PR, Badylak SF, Saris DB, Dhert WJ, Malda J	Review	10.1016/j.tibtech. 2012.12.004	2013	58	369
6	Biomaterials for articular cartilage tissue engineering: Learning from biology	Armiento AR, Stoddart MJ, Alini M, Eglin D	Review	10.1016/j.actbio. 2017.11.021	2018	58	282
7	Cell-laden hydrogels for osteochondral and cartilage tissue engineering	Yang J, Zhang YS, Yue K, Khademhosseini A	Review	10.1016/j.actbio. 2017.01.036	2017	56	335
8	A biomimetic three-dimensional woven composite scaffold for functional tissue engineering of cartilage	Moutos FT, Freed LE, Guilak F	Article	10.1038/nmat1822	2007	51	557
9	Self-assembling peptide hydrogel fosters chondrocyte extracellular matrix production and cell division: implications for cartilage tissue repair	Kisiday J, Jin M, Kurz B, Hung H, Semino C, Zhang S, Grodzinsky AJ	Article	10.1073/pnas.142309999	2002	40	848
10	Hydrogel design for cartilage tissue engineering: a case study with hyaluronic acid	Kim IL, Mauck RL, Burdick JA	Review	10.1016/j.biomaterials. 2011.08.073	2011	40	355

The two other original articles described the application of tissue engineering materials to promote cartilage tissue regeneration. Moutos FT et al. (Moutos et al., 2007) proposed a microscale three-dimensional weaving technique to create anisotropic three-dimensional woven structures. The mechanical properties of the resulting composite graft were found to be similar to those of native articular cartilage through compression, tension, and shear tests, reproducing the anisotropic, viscoelastic, and tension-compression nonlinear initial characteristics of native articular cartilage. Kisiday J et al. (Kisiday et al., 2002) investigated the effect of self-assembling peptide hydrogels on chondrocytes and found that they could promote extracellular matrix generation and cell division in chondrocytes. They also reported that self-assembling peptide hydrogels could induce chondrocyte secretion of cartilage proteins and collagen and improve the morphological characteristics of chondrocytes. Furthermore, the other 6 reviews covered the utilization of biomaterials and biomanufacturing techniques for creating artificial cartilage in cartilage tissue engineering and discussed various biomaterials, including collagen, glycoproteins, polylactic acid, hyaluronic acid and chitosan, and their applications in cartilage tissue engineering are explored.

### 3.8 The evolution keywords and themes

**FIGURE 5:**
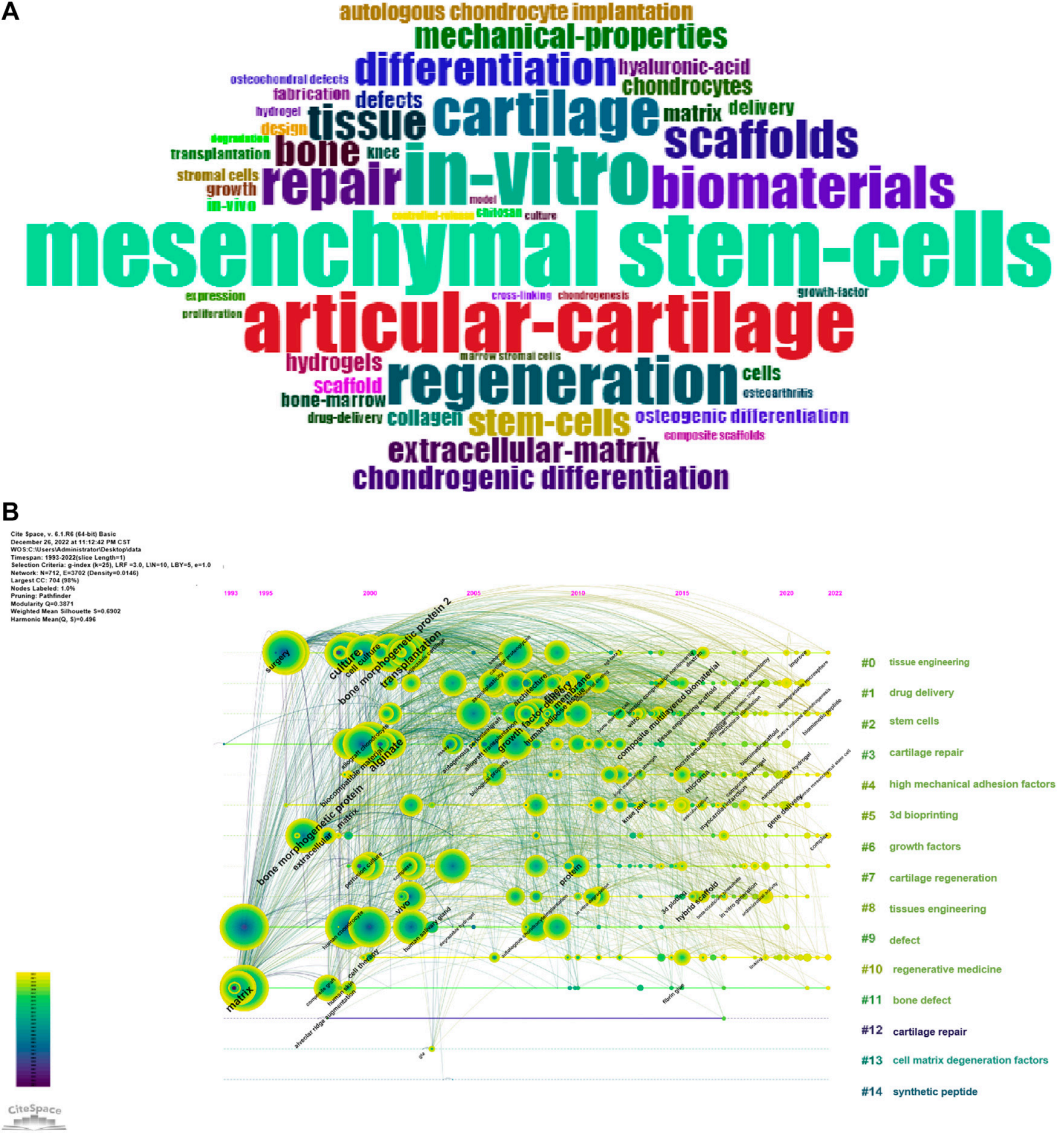
**(A)** Keyword cloud of the top 50 keywords. **(B)** Co-occurrence analysis of keywords and clusters of the searched keywords.

**FIGURE 6:**
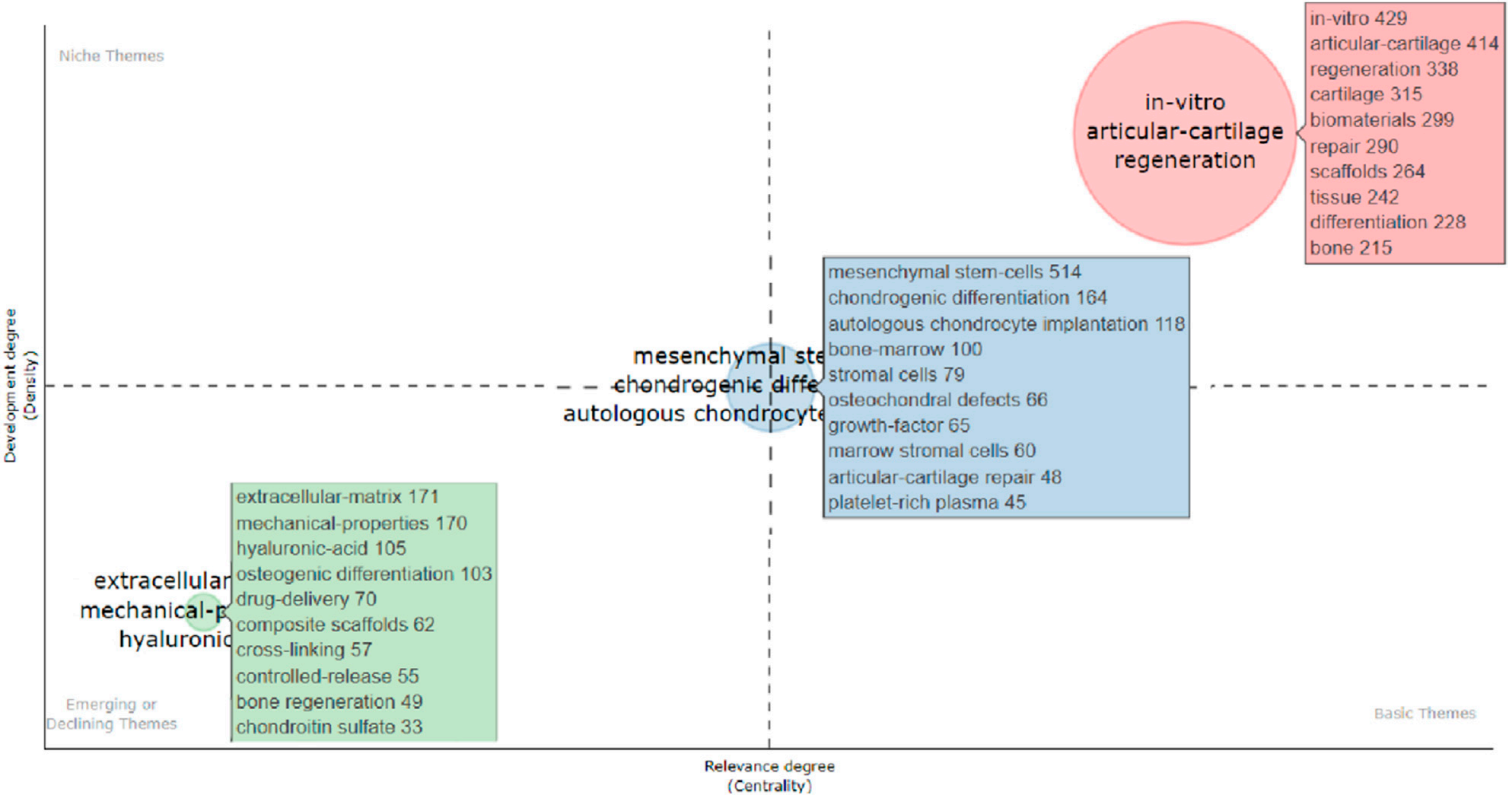
Thematic map illustrating clusters and keywords identified by the co-occurrence network. The X-axis represents the centrality (i.e., the degree of interaction of a network cluster compared with other clusters) and provides information about the importance of a theme. The Y-axis symbolizes the density (i.e., measures the internal strength of a cluster network, which can be assumed to measure the theme’s development). Accordingly, the first quadrant identifies motor themes (i.e., well-developed and important themes for structuring a research field). The second quadrant contains highly developed and isolated themes (i.e., themes of limited importance for the field). The third quadrant comprises emerging or declining themes (i.e., weakly developed and marginal themes). The fourth quadrant includes basic and transversal themes (i.e., general topics that are transversal to different field research areas).

The Bibliometrix R package was used to produce the top fifty keyword clouds (Figure 5A), with larger keywords indicating more frequent occurrences. The top five keywords were: “mesenchymal stem-cells (*n* = 514),” “in-vitro (*n* = 429),” “articular-cartilage (*n* = 414),” “regeneration (*n* = 338)” and “cartilage (*n* = 315).” CiteSpace was used for keyword co-occurrence analysis, and the keywords were clustered. As shown in Figure 5B, there were 14 clusters: “0# tissue engineering; 1# drug delivery; 2# stem cells; 3# cartilage repair; 4# high mechanical adhesion factors; 5# 3d bioprinting; 6# growth factors; 7# cartilage regeneration; 8# tissue engineering; 9# defect; 10# regenerative medicine; 11# defect; 12# cartilage repair; 13# cell matrix degeneration factors; 14# synthetic peptide.” Based on the impact of these 14 clusters on cartilage repair and regeneration, they could be summarized into three themes: (1) “biomaterials and scaffolds;” (2) “Cell type and differentiation;” and (3) “extracellular matrix materials and delivery systems.” Then, the Bibliometrix R package was used to create a thematic map (Figure 6), and the thematic map revealed that the study topics could also be divided into three themes, and according to the displayed keywords, their contents were consistent with the descriptions provided above. “Biomaterials and scaffolds” were located in the first quadrant, the theme “cell type and differentiation” demonstrated average centrality and density, and “Extracellular interstitial materials and delivery systems” topics were in the third quadrant.

## 4 Discussion

In the era of big data, new researchers can rapidly gain insights into a specific research field by performing bibliometric analysis. However, as of yet, no studies have conducted bibliometric analysis regarding cartilage tissue healing and regeneration based on biocompatible materials. Biocompatible materials are becoming more prevalent in regenerative medicine, particularly in the field of improving cartilage tissue healing. To gain insight into the development of cartilage tissue healing using biocompatible materials, we conducted a bibliometric analysis through a thorough literature search to identify relevant articles published between 1993 and 2022, which indicated a significant increase in the number of publications related to this field over the past 2 decades. We also identified the major countries and institutions involved in research on cartilage tissue healing using biocompatible materials and visualized the evolution of hot topics in this area. Additionally, the most frequently used biocompatible materials for cartilage tissue regeneration and their effects on the healing process were identified. Collectively, these findings from the systematic review and bibliometric analysis provide a valuable resource for researchers and clinicians interested in cartilage tissue healing using biocompatible materials.

### 4.1 Current status and major contributing countries

The number of publications is a commonly used metric to gauge the level of interest among researchers in a particular field (Durieux and Gevenois, 2010; Han et al., 2022). In regard to cartilage tissue healing using biocompatible materials, there has been a steady increase in the number of publications over time. The first stage of this research area, spanning from 1993 to 2007, exhibited a relatively stable trend, with fewer than 50 new articles published annually. During this time, most prolific authors were based in the United States. The second stage, from 2013 to 2022, saw a marked increase in the annual number of new publications, with more than 50 per year except for 2009, demonstrating a gradual upward trend. Since then, there has been a substantial increase in the number of publications from China, surpassing that of the United States in 2020. As of 2022, China has become the most productive country in this field, with 1,514 publications. However, the United States has been active relatively early in this field and has collaborated frequently with other countries and regions.

### 4.2 Active institutions and authors

We also identified the most productive institutions in this field. Among the top 10 institutions, five were from China, two were from Portugal, one was from Singapore, and two were from the United Kingdom. Tufts University, Columbia University, and Harvard University were among the institutions that were active in this field earlier. University of Minho, Sun Yat-Sen University, and South China University of Technology became more active after 2015. As of 2020, the majority of the institutions active in this field were Chinese, such as Zhejiang University, Shanghai Jiao Tong University, and Huazhong University of Science and Technology. Due to the possibility of identification errors in the names of authors from China, we did not systematically analyze the contributing authors in this field. It is worth noting that Reis RL from the University of Minho is the most active author in this field, with 52 publications that have been cited 2,597 times and 6 articles having more than 100 citations. Their research focuses on various aspects of tissue regenerative medicine and biomaterials, including developing and applying biomaterials, tissue regenerative medicine, and related research areas. The most cited article is “Gellan gum: a new biomaterial for cartilage tissue engineering applications.” This article discussed the use of bone morphogenetic proteins (BMPs) in bone and cartilage repair. It also described various carriers, such as nanoparticles, 3D scaffolds, membranes and hydrogels that can deliver BMPs, outlined some of the clinical uses of BMPs, such as spinal fusion and long bone defect healing, and noted that BMP-2 and BMP-7 were approved by the US Food and Drug Administration for certain medical cases. Overall, the article suggested that BMPs have significant potential in the field of regenerative medicine (Bessa et al., 2008). In addition, Reis RL et al. (Oliveira et al., 2010) discussed the potential use of gellan gum hydrogels as cell support in cartilage regeneration. The study evaluated the mechanical and structural properties of the hydrogels, as well as their viscoelastic and rheological properties and biological performance, and the results showed that they were non-cytotoxic and could be used to encapsulate and culture human nasal chondrocytes, resulting in normal chondrocyte morphology. Another study examined the physicochemical properties of silk fibroin scaffolds prepared from high-concentration aqueous silk fibroin solutions and reported that the scaffolds had a macro/microporous structure with homogeneous porosity distribution and exhibited concentration-dependence in terms of mechanical properties, water uptake ratio, and stability. The scaffolds were proposed to be suitable for meniscus and cartilage tissue-engineered scaffolding (Yan et al., 2012).

### 4.3 Active journals and co-cited journals

It is important for researchers to identify the leading journals in their field before submitting their work, as top-cited journals can serve as authoritative journals in a given field. “Acta Biomaterialia” ranked as the most productive journal, with 84 publications, while “Biomaterials” ranked the second most productive journal with the highest h-index and total citations (49, 15,300). Based on total citations, the most influential journals in this field include “Biomaterials,” “Acta Biomaterialia,” and “Journal of Biomedical Materials Research Part A.”

### 4.4 Research hotspots and trend

Keyword co-occurrence analysis and co-cited references serve as tools for visualizing the knowledge network and frontier research within a field, as described by Chen et al. (Chen, 2004). In this particular area, research topics can be broadly categorized into three main themes: (1) “biomaterials and scaffolds;” (2) “cell types and differentiation;” and (3) “extracellular matrix materials and delivery systems.”

“Biomaterials and scaffolds” was positioned in the first quadrant, which indicates the most advanced development in this area. Current research on biomaterials used for cartilage tissue healing and regeneration covers a wide range of directions, including hydrogels, protein materials, and nanofibers. Among these, the application of hydrogels in repairing and regenerating cartilage tissue is a particularly active area of study. In addition to their use as scaffolding materials, hydrogels also have the potential as a replacement in cartilage repair surgery (Xue et al., 2014; Shi et al., 2017). Hydrogel is a type of biomaterial with high water absorption and elasticity, making it widely used in the repair and regeneration of cartilage tissue. Some of these common hydrogels include polyethylene glycol (Liu et al., 2021; Song et al., 2021), polyvinyl alcohol (Zhao et al., 2020; Ding et al., 2021), hydroxylated polylactic acid (Shi et al., 2021; Weitkamp et al., 2021; Xin et al., 2022), and chitosan (Mohan et al., 2017; Li et al., 2021). In addition, they can be used as a matrix for culturing chondrocytes for cartilage regeneration *in vitro* (Kisiday et al., 2002; Kim et al., 2011; Shin et al., 2012). In a study conducted by J Kisiday et al. (Kisiday et al., 2002), a self-assembled peptide hydrogel was developed as a scaffold for articular cartilage repair. The study demonstrated that chondrocytes seeded within peptide hydrogels retained their morphology and formed a cartilage-like extracellular matrix rich in proteoglycans and collagen II. Additionally, the accumulation of extracellular matrix was shown to parallel increases in material stiffness, indicating the formation of mechanofunctional nascent tissue. Overall, this research highlights the potential of self-assembled peptide hydrogels as scaffolds for synthesizing and accumulating *bona fide* cartilage-like extracellular matrix for cartilage tissue repair.

In addition, researchers have tried to optimize the physical properties of hydrogels. Sun et al. (Sun et al., 2013) discussed the potential of polyampholyte hydrogels as biomaterials due to their ability to combine multiple mechanical properties such as stiffness, strength, toughness, damping, fatigue resistance and self-healing with biocompatibility. They noted that the randomness of the ionic bonds supported the hydrogel’s mechanical properties to be tuned over a wide range. This approach is simple and could open up new possibilities for using tough hydrogels in various applications.

In their search for specific materials for cartilage repair and regeneration, researchers have investigated the use of graphene as a biocompatible scaffold for tissue engineering applications. Nayak et al. (2011) found that graphene did not hinder the proliferation of human mesenchymal stem cells and even accelerated their differentiation into bone cells. In addition, microporous bacterial cellulose scaffolds have been shown to have good mechanical properties and to support the formation of denser mineral deposits (Zaborowska et al., 2010). Furthermore, collagen-hyaluronic acid-hydroxyapatite-halite nanotube-single-walled carbon nanotube composites have demonstrated potential as biomaterials for cartilage regeneration (Zhang et al., 2022).

The theme “Cell type and differentiation” showed average centrality and density. The success of cartilage tissue repair and regeneration is heavily influenced by the types of cells used and their differentiation status (Kangari et al., 2020; Guo et al., 2022; Wang et al., 2022). Many researchers have explored the potential of mesenchymal stem cells (MSCs) as a solution for cartilage regeneration. MSCs are pluripotent adult stem cells found in various tissues, such as bone marrow (Rodríguez-Lozano et al., 2020), adipose tissue (Peng et al., 2017), synovial membrane (Kondo et al., 2019), and umbilical cord Wharton’s jelly (Grau-Vorster et al., 2019). They can self-renew, differentiate into multiple cell types, and regulate the immune system, making them a promising candidate for cartilage regeneration (Maheshwer et al., 2021). According to a meta-analysis by Hirotaka Iijima (Iijima et al., 2018), the use of MSCs in clinical settings appears to be generally safe, with no serious adverse events reported. Injecting MSCs directly into the joint or using arthroscopic implantation has been shown to significantly reduce pain and improve knee function. In addition, MSCs have been demonstrated to have the potential to differentiate into cartilage tissue and produce extracellular matrices that are important for the recovery of cartilage function (Ma et al., 2018; Le et al., 2020). By releasing various cytokines, growth factors, and chemokines at the targeted repair areas, MSCs can create a regenerative microenvironment and aid in the repair of injured cartilage (Mastbergen et al., 2013; Han et al., 2019; Le et al., 2020). Biomaterials can serve as scaffolds or substrates for cell adhesion and differentiation and can also influence the microenvironment of cell culture. MSC-based therapies, which involve combining MSCs with exogenous stimuli and engineered scaffolds, have demonstrated significant advances in cartilage regeneration. Collagen, a major component of the cartilage extracellular matrix, has been found to promote MSC proliferation, improve matrix formation, and inhibit hypertrophy of chondrocytes derived from BSCs (Chiu et al., 2014; Xia et al., 2018). Clinical investigations into the use of collagen for cartilage repair have also been conducted, with autologous MSCs and collagen scaffolds utilized to repair torn meniscal cartilage. A study involving five patients who were followed up for 2 years showed significant improvement in clinical symptoms (Whitehouse et al., 2017). Furthermore, protein scaffolds made from fibrin have been shown to promote MSC proliferation (Liu et al., 2018). Fibrin-based hydrogel-encapsulated BM-MSCs have been found to induce BM-MSC chondrogenic differentiation, suggesting that fibrin may be a suitable encapsulating matrix for the cartilage phase in an osteochondral construct (Ho et al., 2010).

The theme “Extracellular Interstitial Materials and Delivery Systems” is positioned in the third quadrant, indicating a fading or emerging theme. Research on extracellular matrix materials, which are highly biocompatible and can be used to repair or regenerate cartilage, has gradually declined. These materials are typically extracted from the body’s extracellular matrix such as collagen, elastin, and fibrous tissue. Related research in this field aims to develop an optimal scaffold for cartilage regeneration by combining natural and artificial materials (Zhang et al., 2019). While synthetic polymers can be used to customize hybrid scaffolds with essential mechanical properties and structures, the addition of natural polymers can provide bioactive molecules. Researchers have investigated the effects of different extracellular matrix contents on the differentiation of bone marrow mesenchymal stem cells. They discuss several studies that utilize PEG hydrogels containing Col or HA (Hwang et al., 2011), PCL microfibers coated with acellular cartilage ECM (Liao et al., 2010), nanoscale hydroxyapatite loaded on PLLA fibers (Spadaccio et al., 2009), and hybrid scaffolds consisting of PGA-hydroxyapatite and autologous BM-MSCs to investigate the effects of these materials on chondrogenic differentiation of BM-MSCs and the repair of osteochondral defects (Zhou et al., 2008). These findings suggest that the ECM content in these materials can influence the differentiation of BM-MSCs and the repair of osteochondral defects.

The study of exosomes as delivery tools has become a hot topic in the field of cartilage regeneration. Exosomes are small vesicles with diameters of approximately 30–200 nm that can transport a variety of bioactive molecules, such as miRNAs, proteins, and small molecules, to target cells through their bilayer phospholipid structure (Marote et al., 2016; McGough and Vincent, 2016), making them widely considered as an ideal platform for targeted drug delivery (Katare et al., 2014; Whiteside, 2016). By encapsulating therapeutic agents within exosomes, it is possible to selectively deliver them to specific cells or tissues, potentially reducing off-target effects and improving treatment outcomes (Katare et al., 2014; Whiteside, 2016; Pegtel and Gould, 2019; Yao et al., 2019). In a recent study, Xu *et al.* (2021) investigated the potential of exosomes with engineered MSC-binding peptides in delivering a small molecule called Kartogenin (KGN) to synovial fluid-derived mesenchymal stem cells (SF-MSCs) to induce chondrogenesis. Their results suggested that KGN delivered by engineered exosomes could efficiently enter SF-MSCs, thereby increasing its effective concentration within the cell and strongly promoting the chondrogenesis of SF-MSCs both *in vitro* and *in vivo*. Thus, their delivery method holds great potential as an advanced stem cell therapy for osteoarthritis. Another study investigated the role of exosomal circRNAs in osteoarthritis and found that overexpression of exosomal circRNAs 0001236 alleviated cartilage degradation and inhibited OA progression, providing a potential therapeutic strategy for the treatment of OA (Mao et al., 2021). By investigating the mechanism of action of MSC exosome-regulated cellular processes and exosome-mediated cartilage repair responses, they reported that exosomes positively affected cell proliferation, infiltration and matrix synthesis and were associated with regenerative immunophenotype. The increased cell proliferation and infiltration were attributed to adenosine activation of AKT and ERK signaling, which was mediated by exosomal CD73. The regenerative immunophenotype was characterized by a higher infiltration of CD86^+^ M1 macrophages and CD163+ regenerating M2 macrophages, along with a decrease in proinflammatory synovial cytokines IL-1β and TNF-α. Collectively, these findings demonstrate that MSC exosomes may effectively repair critical-sized osteochondral defects by mobilizing multiple cell types and activating several cellular processes (Zhang et al., 2018; Zhang et al., 2019; Chew et al., 2019).

## 5 Limitation

This bibliometric analysis has several limitations that should be considered. First, the database used may not be completely up-to-date, which could result in selection bias in the literature retrieved. Second, the study only used the Web of Science core collection-EXPANDED database as a data source, and other databases such as PubMed, Scopus, and Google Scholar may provide more comprehensive results. Third, the study only employed two indicators (CPP and TC) to measure academic influence, and other measures such as the G-index, H-index, SJR, CiteScore, and SNIP could also be useful in assessing the quality of publications or journals. Fourth, the study only included English literature and did not consider non-English publications, which could potentially impact the results. Finally, the study did not analyze or compare the funding of different studies, which could also be a factor in the academic influence of a particular research topic.

## 6 Conclusion

This study used bibliometric analysis to investigate the development of cartilage tissue healing using biocompatible materials. The findings revealed a significant increase in related publications over the past 2 decades, with China being the most productive country in this field. The study identified active institutions, authors, journals, and co-cited journals. Keyword co-occurrence analysis and co-cited references were utilized to visualize the knowledge network and frontier research in this field. The research topics were classified into three main categories: biomaterials and scaffolds, cell type and differentiation, and extracellular matrix materials and delivery systems. Hydrogels and MSCs were commonly used in cartilage tissue healing and regeneration. Exosome-based delivery systems emerged as a research hotspot in this field. Although this study has limitations, the bibliometric analysis still provides valuable insights into the development of cartilage tissue healing using biocompatible materials.

## Data Availability

The original contributions presented in the study are included in the article/Supplementary material, further inquiries can be directed to the corresponding authors.
